# Stress System Dynamics during “Life As It Is Lived”: An Integrative Single-Case Study on a Healthy Woman

**DOI:** 10.1371/journal.pone.0029415

**Published:** 2012-03-05

**Authors:** Christian Schubert, Willi Geser, Bianca Noisternig, Dietmar Fuchs, Natalie Welzenbach, Paul König, Gerhard Schüßler, Francisco M. Ocaña-Peinado, Astrid Lampe

**Affiliations:** 1 Clinical Department of Medical Psychology, Innsbruck Medical University, Innsbruck, Austria; 2 Institute of Psychology, University Innsbruck, Innsbruck, Austria; 3 Division of Biological Chemistry, Biocentre, Innsbruck Medical University, Innsbruck, Austria; 4 Clinical Department of Internal Medicine, Innsbruck Medical University, Innsbruck, Austria; 5 Department of Statistics and Operations Research, University of Granada, Granada, Spain; Wayne State University, United States of America

## Abstract

Little is known about the dynamic characteristics of stress system activity during “life as it is lived”. Using as representative a study design as possible, this investigation sought to gain insights into this area. A healthy 25-year-old woman collected her entire urine over a period of 63 days in 12-h intervals (126 measurements) to determine cortisol and neopterin (immune activation marker) levels. In addition, she filled out questionnaires on emotional state and daily routine in 12-h intervals, and was interviewed weekly to identify emotionally negative and positive everyday incidents. Adjusted cross-correlational analyses revealed that stressful incidents were associated with cyclic response patterns in both urinary cortisol and urinary neopterin concentrations. Urinary cortisol levels first decreased 12–24 h after stressful incidents occurred (lag 1: −.178; *p* = 0.048) and then increased a total of 72–84 h later (lag 6: +.224; *p* = 0.013). Urinary neopterin levels first increased 0–12 h before the occurrence of stressful incidents (−lag 1: +.185; *p* = 0.040) and then decreased a total of 48–60 h following such stressors (lag 4: −.181; *p* = 0.044). Decreases in urinary neopterin levels were also found 24–36 and 48–60 h after increases in pensiveness (lag 2: −.215; *p* = 0.017) and depressiveness (lag 4: −.221; *p* = 0.014), respectively. Findings on emotionally positive incidents sharply contrasted with those dealing with negative experiences. Positive incidents were followed first by urinary cortisol concentration increases within 12 h (lag 0: +.290; *p* = 0.001) and then by decreases after a total of 60–72 h (lag 5: −.186; *p* = 0.039). Urinary neopterin levels first decreased 12–24 h before positive incidents occurred (−lag 2: −.233; *p* = 0.010) and then increased a total of 12–24 h following these incidents (lag 1: +.222; *p* = 0.014). As with previous investigations on patients with systemic lupus erythematosus (SLE), this study showed that stress system response can be considerably longer and more complex and differentiated than findings from conventional group studies have suggested. Further integrative single-case studies will need to be conducted in order to draw firm conclusions about stress system dynamics under real-life conditions.

## Introduction

The hypothalamus-pituitary-adrenal (HPA) axis is a key mediator between the natural environment and the organism, and serves to maintain inner equilibrium [1], [2]. This adaptational task is performed by interacting subsystems, self-sustained oscillators and multiple feedback loops that react to internal and external inputs [2]–[4]. Such complex organization results in strong temporal fluctuations in the release of cortisol, the main effector molecule of the HPA axis [5]–[7]. There is ample evidence that the HPA axis is connected to a variety of other physiological systems, such as the gonadal, growth, thyroid and immune systems [1], and that its interconnectedness with the immune system is crucial in stress-mediated illness [8].

Although over the past decades tremendous efforts have been made to empirically describe the adaptational activity of the stress system to psychosocial stressors, relatively few conclusions can be drawn from this literature [9]–[13]. One of the major shortcomings has been the insufficient knowledge concerning the dynamic characteristics of the stress process, e.g. the duration and timing of stress system response [12] and its time-dependent patterns [11].

As to the time kinetics of stress-induced cortisol concentration changes, laboratory studies in healthy individuals have revealed that cortisol levels generally increase 20–40 min following acute stressors and return to baseline after a total of about 1 h [9], [14], [15]. On the other hand, changes in concentrations of cytokine interleukin-6 (IL-6) in response to acute stressors may follow alterations in the sympathetic nervous system (SNS) and in HPA axis activity with a temporal delay [16]. Specifically, plasma concentrations of the cellular immune parameters IL-6, tumor necrosis factor alpha (TNF α) and interleukin-1 receptor antagonist (IL-1Ra) rise between 30 min and 2 h following standardized behavioral tasks [17]–[19]. Moreover, a recent study has demonstrated increased mononuclear cell interleukin-1beta (IL-1β) gene expression at 30, 75 and 120 min post-stress [20]. However, how long stress-induced inflammatory parameter increases endure and when they return to baseline after the onset of acute stressors has not yet been established [12].

There are indications in healthy individuals that psychoendocrine stress responses are closely coupled over time [21] and that the pattern of stress system responses may be biphasic or cyclic. Miller et al. [11] suggest that at the beginning of chronic stress, adrenocorticotropin (ACTH) and cortisol concentrations are elevated and that, with time, cortisol secretion returns to below normal. Regarding immune activity, studies have shown that circulating counts and lytic activity of natural killer cells first increase concomitantly in response to stressors and then decrease within 2 h [22]–[24].

To date, only a few studies have investigated long-term stress system effects in response to acute laboratory stressors or to the administration of stress hormones. Such studies show that the duration of the stress process is considerably longer than short-term laboratory studies in stress research have suggested [25], [26]. Seligman et al. [27], for example, showed that the brain noradrenergic system of rats exposed to inescapable shocks (IS) was suppressed for a period of 48–72 h. Moreover, exposure of rats to a single session of IS resulted in an increase in basal total corticosterone that persisted 48–96 h after IS termination [28]. In healthy humans, Posener et al. [29] demonstrated that corticotropin-releasing hormone (CRH) and possibly ACTH inhibit ACTH secretion 21 and 45 h following hormone administration.

This conflicting evidence on the dynamic characteristics of the stress process confirms that, with regard to everyday stressors, the representativeness of laboratory stress tasks is sometimes low [30]–[34] and that the testing of stress system functioning should ideally be based on real-life conditions rather than laboratory settings [35]. However, investigating the temporal characteristics of the stress process under real-life conditions implies methodological requirements for the assessment and analysis of time series data and emotionally relevant everyday incidents – requirements that have only recently begun to be considered in stress research.

In so-called ecological momentary assessment (EMA) or experience sampling method (ESM) studies, subjects gather psychological data (i.e. daily events, feelings) and collect saliva samples on a momentary basis each day over a period of several days or weeks. Such naturalistic studies often apply stepwise multiple regression analysis and multilevel growth-curve modeling for statistical analysis. They have shown temporal intervals between the occurrence of everyday experiences and stress system activity changes ranging from 1 day for cortisol [36]–[38] to 1–2 days for immune parameters such as secretory immunoglobulin A (sIgA) antibody levels [39]. Although this kind of study design is a great step forward in terms of ecological validity in stress research, EMA or ESM studies may nevertheless lack representativeness due to, for one, the programmed wristwatches used in such studies, which produce acoustic signals that disrupt everyday activities. Also, the irregular serial measurements and the exclusion of nighttime data lead to incomplete serial data sets that are not appropriate for time-series analysis. The exclusive use of questionnaires and/or diaries, moreover, means that information on the personal meaning of incidents may remain unexplored [40]–[42].

Our investigations of stress under real-life conditions have been based on an “integrative” research approach that aims to assess, to the greatest extent possible, “life as it is lived” [43]. This approach is designed to consider the continuity and openness of an ever-changing everyday reality, in which personally meaningful incidents are often distributed unpredictably in time. Integrative single-case studies combine quantitative and qualitative methods with a specific focus on: i) the dynamic interdependencies between psychosocial, psychological and physiological variables based on a large number of serial measurements and time-series analysis, and ii) the emotional meaning or personal significance of daily incidents through in-depth interviews and hermeneutic analysis of interview data [44].

The first integrative single-case studies were conducted with patients with systemic lupus erythematosus (SLE). SLE is a chronic autoimmune disease with established HPA axis dysfunction and hypocortisolism [8], [45], [46]. In these studies, patients collected their daily urine, and urinary cortisol and neopterin concentrations were measured to monitor stress system activity non-invasively. Neopterin is released by human monocyte-derived macrophages and dendritic cells in large amounts, preferentially upon stimulation with interferon-gamma (IFN-γ), a pro-inflammatory cytokine reflecting the T helper type 1 (Th1) immune activation status [47].

Using Auto-Regressive-Integrated-Moving-Average (ARIMA) modeling of time series and adjusted cross-correlational analyses, our studies on SLE patients have consistently shown that the stress reaction process is associated with cyclic or biphasic stress system responses that remain evident for days. Specifically, whenever patients experienced emotionally negative stressful incidents, urinary cortisol first increased within 24–36 h and then decreased after a total of 36–48 h, whereas urinary neopterin first decreased after 36–48 h and then increased after a total of 60–72 h [48], [49]. Moreover, biochemical changes in response to emotionally pleasing incidents contrasted sharply with these findings. Here, urinary cortisol first decreased after 36–48 h and then increased after a total of 48–60 h, whereas urinary neopterin first increased after 24–36 h and then decreased after a total of 84–96 h [50].

The present study tested stress-system functioning (cortisol, neopterin) under everyday-life conditions in a healthy woman. Emotionally stressful incidents and emotionally pleasing incidents were investigated as possible triggers of HPA axis activity. The following questions were posed: 1) How long are the time intervals between the onset of stress and stress system response? 2) Are there cyclic patterns of stress system parameter changes in response to emotionally meaningful incidents? 3) Do negative incidents differ from positive incidents in terms of their effects on stress system functioning? 4) Does the functioning of a healthy stress system differ from that found in our studies on patients with SLE?

## Methods

### Study design

The subject was interviewed one month prior to the study to determine stressful life events and chronic difficulties during the preceding two years. Shortly before the start of the study, she was given a thorough physical examination and psychiatric evaluation (Diagnostic and Statistical Manual of Mental Disorders IV, DSM-IV) [51]. During the study period of 63 days (December 16^th^, 1999 to February 16^th^, 2000), data were gathered in 12-h intervals (total: 126 time intervals) during normal daily activities.

In order to ensure study representativeness, it was critical to interfere as little as possible with the subject's normal routine and to attain complete data sets without temporal gaps. The biochemical data were obtained from the subject's urine. On each day of the study period, the subject collected her entire urine in day (from 08:00–20:00 h) and night portions (from 20:00–08:00 h). She recorded the volume of the collected urine and froze several urine aliquots from each 12-h portion at −20°C. Each week, the subject brought these frozen samples to the laboratory, where they were stored at −70°C. She was examined weekly to monitor general health. All biochemical analyses were conducted following the study period.

Psychosocial data were obtained from questionnaires. Twice each day, at the end of each 12-h interval (at 20:00 h and at 08:00 h), the subject answered questions about emotional states, daily life-style factors (alcohol consumption, medication, etc.), and potential signs of infection. In addition, she took notes on all psychosocial incidents (positive and negative) during the preceding 12 h. Before the study began, the subject received thorough instruction in all the tasks involved in the study. She was asked to complete the daily study tasks in quiet surroundings, without any interruption.

The psychosocial significance of the recorded incidents was determined in weekly interviews and rated by a three-person panel following the study period. Interviewer and raters were blind to the physiological data.

The subject fulfilled the following inclusion criteria: healthy, age after puberty and before menopause, native German speaker, residence in or near Innsbruck, Austria. Pregnancy was an exclusion criterion in this study.

The subject gave informed consent in written form to her participation and to the publication of data. The Ethics Committee of the Medical Faculty of the University of Innsbruck approved the design. The subject received 11,150 Austrian Schillings (approx. 800 Euro) for her participation.

### Subject description

#### Anamnestic information

The subject of this study is a healthy 25-year-old woman (height: 1.80 m; weight: 74 kg; Body Mass Index: 22.84). She is a non-smoker and does not use oral contraceptives. Her only surgical history was a tonsillectomy at the age of eight. Four months before the study began, she had suffered from unexplained diarrhea for approx. six weeks after returning from a trip to Africa.

#### Biographical sketch

The subject's parents broke up shortly before her birth. Until the age of five, the subject was raised in large part by her grandparents and her aunt, while her mother (45 y.o. at study start) was at work. During this time, she saw her mother only on weekends, if at all. The subject had had face-to-face contact with her biological father (45 y.o. at study start) only once (about one year before study start). She also had rare telephone conversations with him, mostly concerning financial issues surrounding her studies. When the subject was five years old, her mother, who was pregnant with twins, married her first husband (53 y.o. at study start) and moved with her daughter to his home. The twins died shortly after birth. During the following eleven years, four half-brothers were born. The subject finished school at the age of 18 and moved to Innsbruck to study biology at the university. During the study period, she worked part-time at the university as a researcher (10 h per week) and took teacher training courses (6–10 h per week). The subject is single and lives in Innsbruck in a studio apartment. A six-year relationship with a man ended three years before the study start. The subject sees her family every three weeks and her best friend every two or three days. She reports on four other good friends as well as four confidants.

### Psychological measurements

#### Major life events and chronic difficulties

The Life Event and Difficulty Schedule (LEDS) [52] is a semi-structured interview that assesses major life events and chronic difficulties. It requires approx. 2 h to complete. The LEDS was conducted with the subject one month before the study and covered the preceding two years. In addition to its psychodiagnostic character, the LEDS was helpful in learning about the subject and her psychosocial background. This information was useful when conducting the weekly interviews and rating daily incidents.

#### Weekly interviews and rating of emotionally meaningful incidents

The weekly interviews conducted with the subject were semi-structured, consisting of two parts. First, the preceding week's emotionally *negative* or stressful incidents were identified using the Incidents and Hassles Inventory (IHI, Brown and Harris in [44]) – a standardized list of 39 items dealing with common everyday stressors. Examples of items covered by the IHI are “argument/scene with partner” and “criticism/argument at work”. Second, *positive* as well as *negative* incidents recorded by the subject in her daily notes during the previous week were discussed (see Daily Inventory of Activity, Routine and Illness, DIARI). In both parts of the interview, all incidents were examined in detail with the subject, exploring what she thought and did as incidents unfolded, her emotional response, the temporal specifics of each incident, the extent to which incidents were anticipated, persons involved, memories evoked, etc. Each weekly interview lasted approx. one hour and was recorded on audio cassette.

The rating of incidents was performed by three raters and was based on consensus [52]. In one type of analysis, according to the IHI procedure, the rater panel subcategorized emotionally *negative* incidents according to the *degree* of psychosocial stress. Each daily incident was assigned a distinct score on a three-point scale of stress intensity (1 = marked, 2 = moderate, 3 = somewhat), regardless of thematic content. A second analysis of incidents, drawing on a previous integrative single-case study on a patient with SLE [49], [50], subcategorized emotionally *positive* and *negative* incidents according to *thematic content*, whereby each daily incident was assigned to a personally meaningful theme or conflict (e.g. tense relationship with partner), regardless of emotional intensity. This approach provides access to a broader spectrum of psychosocial incidents than can be covered by a standardized item list.

Using the data obtained from the two types of analysis (i.e. degree of stress, thematic content), we constructed time series of incidents by coding all 12-h units containing an incident with “1” and all others with “0”. If more than one meaningful incident occurred within a 12-h unit, we relied on a threshold model of stress and considered only the incident with the highest intensity for time series construction instead of adding incidents of various intensities and qualities (additive model of stress). We did not apply an additive model of stress as we do not know which level of measurement the scores from our incident ratings correspond to – ordinary or higher scale [53]. In other words, it is not clear which algorithm should be used when we want to add up incident scores. Literature on this topic is unfortunately not yet developed enough to deal with such issues appropriately. Thus, in the absence of specific suggestions from other studies of daily incidents, we chose the more parsimonious of the two models of life stress measurement, the threshold model which is the typical approach in investigator-based interview studies dealing with major life events [54], [55].

#### Emotional states

The Eigenschaftswörterliste 60-S (EWL 60-S) [56] is a paper-and-pencil test (in German) that measures emotional state in six categories (mental energy levels, general lethargy, extraversion/introversion, well-being, irritation, anxiousness/depressiveness) using a total of 60 adjectives, each of which is assessed on a four-point scale. Each emotionally negative category consists of twelve adjectives, and each emotionally positive category consists of eight adjectives. The six main categories can also be divided into subcategories. For example, anxiousness/depressiveness is subcategorized into anxiousness, depressiveness, and pensiveness. The EWL 60-S is recommended for use in longitudinal designs. In our study, the EWL 60-S was conducted every 12 h by the subject. Chronbach's alpha for this test is between 0.4 and 0.86 [57]. The test requires approx. 5 min to complete.

#### Daily routine

Using the Daily Inventory of Activity, Routine and Illness (DIARI) [44], a paper-and-pencil test, the subject recorded alcohol and coffee consumption, drug use/medication, sleep, body temperature and any signs of a cold, flu, etc. The subject also rated her amount of physical activity, menstrual flow and menstrual pain during the preceding 12 h using 100 mm visual analogue scales (VAS). Finally, she took notes on all positive and negative incidents, even minor ones. The DIARI requires approx. 5 min to fill out. In this study, the subject completed the DIARI twice each day. Following the study, DIARI data were used to construct a variety of time series.

### Health check

The “Check-up für Normalpersonen” [58] was used to determine whether the subject was ill, had ever suffered from a disease or had needed surgery. The questionnaire contains 33 items and covers all major fields of medicine, including complaints that may be related to psychological/psychiatric disease (e.g. depression, anxiety). Medication, illnesses in the family, alcohol consumption and smoking habits are also surveyed.

### Biochemical analyses

12-h urinary cortisol levels were determined using Radio-Immuno-Assay (RIA) (IBL, Hamburg), and 12-h urinary neopterin levels were determined using High Pressure Liquid Chromatography (HPLC) (Model LC 550; Varian Associates, Palo Alto, CA) as previously described [59]. All 126 urine aliquots were measured in one single run within three months following collection. A new aliquot was used for each of at least three independent determinations. In order to compensate for variations in urine density, urinary cortisol and urinary neopterin concentrations are expressed in micromolar per molar (µmol/mol) creatinine.

### Statistical analyses

Time series (i.e. sequences of *N* data points) were analyzed using SPSS-Trends™ 14.0 [60]. In order to determine whether one variable significantly preceded and thus predicted another, cross-correlations between the variables under study were computed, both at lag 0 (i.e. contemporaneous correlation) and at higher lags, i.e. up to +/−7. We considered positive and negative lags in the analysis of stress system dynamics because we assume not only that incidents can precede biochemical parameter changes but also that biochemical parameter changes can precede the occurrence of incidents, e.g. due to anticipation [49], [50].

For pairwise computation, SPSS-Trends™ 14.0 [60] includes automatic alpha adjustment. For multiple cross-correlations, however, no alpha adjustment was made in this study. We base this statistical strategy on the idea that integrative single-case studies, at the current stage of research, have a strong exploratory character, thus rendering multiple test adjustments a potential hindrance (as they increase the chances of making a type II error), when in fact flexible approaches are needed for design and analysis [61], [62]. Moreover, critics of multiple test adjustment generally argue that research should not only focus on statistical significance – including related issues such as alpha adjustment – but also on research quality, amplitude of findings (i.e. effect size), and patterns of findings [63], [64].

Nevertheless, this study used adjusted analyses to avoid spurious significances in cross-correlation functions. Time series in psychobiological research are typically governed by two main factors: an internal dynamic structure, giving rise to serial dependency (e.g. diurnal rhythm in cortisol levels), and serially uncorrelated disturbances. Because of these serial dependencies, unadjusted cross-correlational analysis between psychophysiological variables may lead to spurious (positive or negative) cross-correlations arising solely from autocorrelations within the two series. Therefore, we separated the serial dependencies from the disturbances by applying ARIMA (Auto-Regressive-Integrated-Moving-Average) filters for each time series. As ARIMA modeling assumes stationarity, we took differences in the time series when the mean of a time series was not constant, and we log-transformed a time series when the variance in a time series had to be stabilized [41], [65]. The serial independence of the filtered residuals was tested using the Ljung-Box test, with up to 4 lags [66]. Adjusted cross-correlational analyses with the filtered residuals were then calculated to determine whether the disturbances driving the various time series were significantly correlated. In this study, asymptotic p-values were computed for the cross-correlation functions, which is in accordance with the assumptions of Bartlett [67] and Brockwell and Davis [68]. Cross-correlations were considered statistically significant when they met the *p*<.05 criterion.

## Results

The LEDS interview and rating revealed that two years preceding the study the subject experienced five stressful life events (three rated as “moderate”, two as “somewhat”) and three chronic difficulties (one rated as “low moderate”, two as “mild”), two of which, i.e. parents' marriage problems (“low moderate”) and mother's health problems (“mild”), were important throughout the whole two-year period.

**Figure 1:**
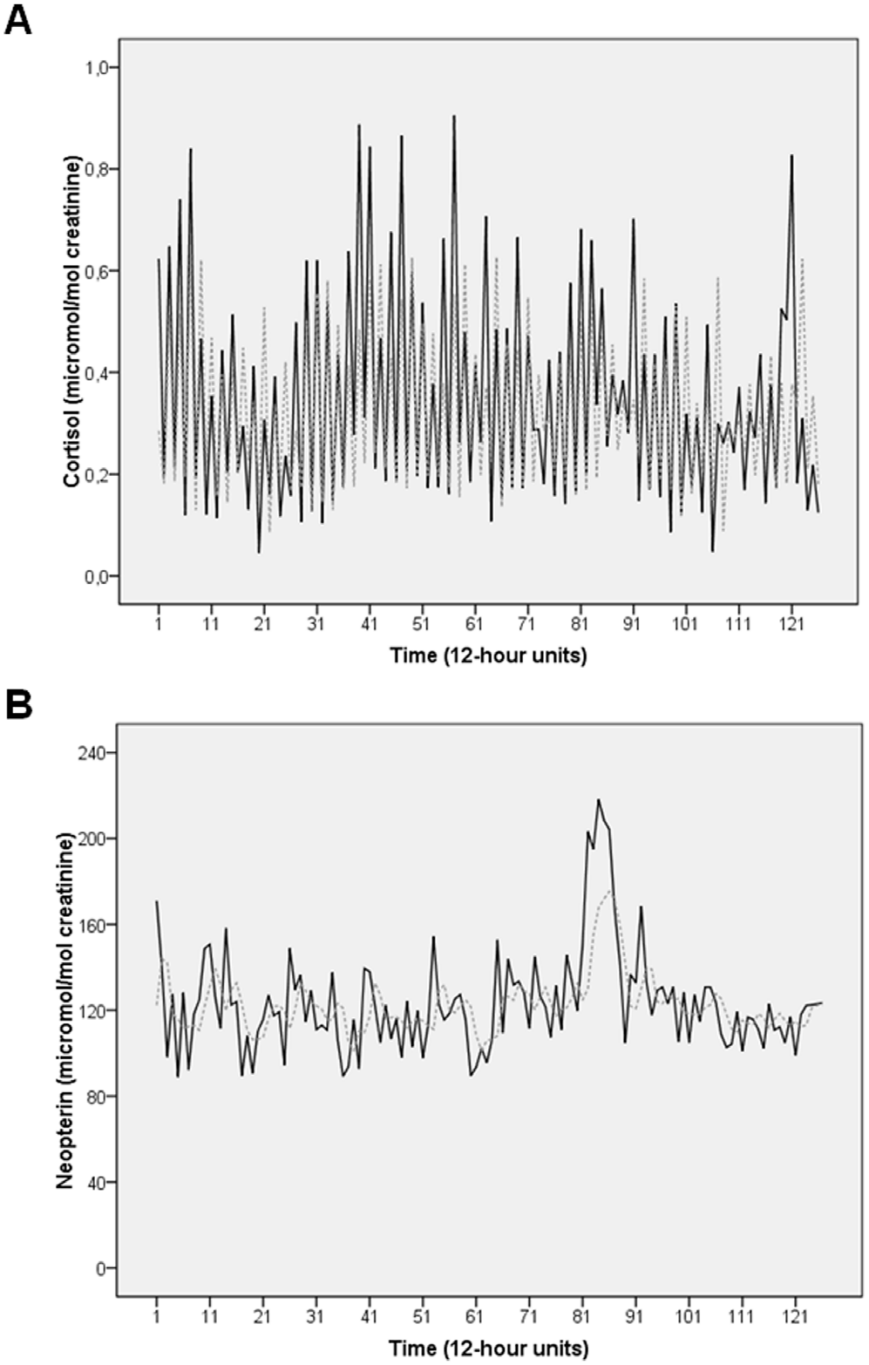
Time series of urinary cortisol and urinary neopterin levels of the healthy woman under study. **A**) shows the time series of urinary cortisol levels, and **B**) shows the time series of urinary neopterin levels. Both the raw data (solid black) and the fit from the ARIMA model representing the series' internal structure (dotted grey) are plotted. Time series cover a period of 63 days or 126 12-hour time-units during which the subject collected her entire urine in day portions (08:00–20:00 h, uneven numbers) and night portions (20:00–08:00 h, even numbers).

Figure 1 shows the time series of urinary cortisol (A) and urinary neopterin (B) from the prospective part of this study (126 consecutive measurements). The urinary cortisol time series shows a typical day-night rhythm of cortisol release (high during day, low at night) best described by a (2,0,0) ARIMA model. The urinary neopterin time series also corresponds to a (2,0,0) model, but with higher neopterin levels at night. The subject's mean urinary cortisol concentration over the whole observation period (*N* = 126) was 0.35±0.21 µmol/mol creatinine, ranging from 0.05 to 0.91. Her mean urinary neopterin concentration was 124±24 µmol/mol creatinine, ranging from 89 to 218. The corresponding absolute values were 2.95±1.77 (0.23–8.44) for urinary cortisol and 1180±672 (292–3974) for urinary neopterin. This study had no missing data.

First, we examined the impact of emotionally negative incidents on the course of urinary cortisol and urinary neopterin levels. The assessment of psychosocial stress according to degree revealed one “marked stressful”, five “moderately stressful”, and 38 “somewhat stressful” incidents during the study period. As this study was not based on an additive model of stress, the six “somewhat stressful” incidents co-occurring with other “somewhat stressful” or “moderately stressful” incidents during the same 12-h unit were not included when constructing the time series; in this study, however, no 12-h unit showed more than two incidents. The final time series of “somewhat, moderately, and marked stressful” incidents thus consisted of a total of 38 incidents. In Table 1, examples of a “somewhat”, a “moderately” and a “marked” stressful incident are described in brief.

**Table 1:** Examples of “somewhat, moderately, and marked stressful” daily incidents.

**Day 15 (time unit 31): Waiting in vain for friends ** ***(somewhat stressful)***
The subject was supposed to meet a friend in a shopping center. However, the subject and her friend waited for each other in different coffee houses. After waiting for 30 minutes, the subject went home feeling a bit sad and exhausted from shopping. She felt even more disappointed when she found out later that another friend had come along, whom she likewise did not meet.
**Day 46 (time unit 91): The subject's stepfather attacks her mother verbally ** ***(moderately stressful)***
The subject spent the weekend in her hometown. During her stay, her mother and stepfather had an argument in which the subject was involved. The stepfather started attacking the subject's mother verbally. The subject was quite angry with her stepfather for half an hour and then sad for another half an hour that he had used such derogatory language to attack her mother.
**Day 61 (time unit 121): Presentation in English at a scientific meeting ** ***(marked stressful)***
The subject, a doctoral student, held a 15-minute presentation in English in the late afternoon at a small scientific meeting which takes place every year near Innsbruck. It was only the second presentation of this kind for her. She had been preparing for weeks, still practicing on the day of the presentation for several hours in front of her room-mate. She was very nervous.

**Figure 2:**
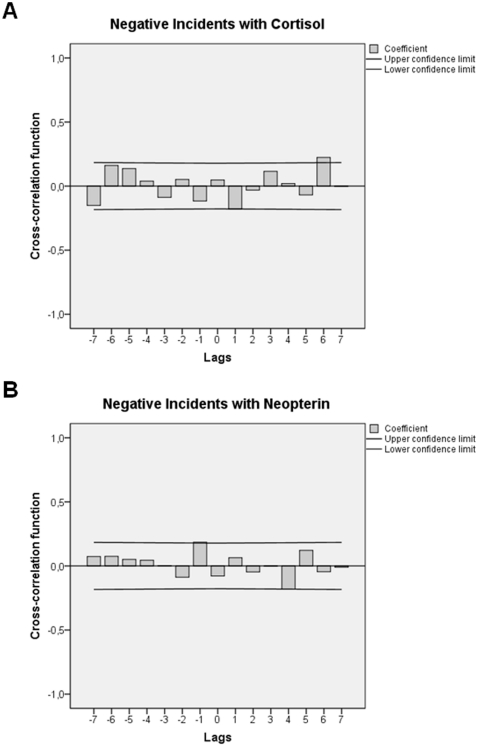
Adjusted cross-correlation functions indicating that negative (“somewhat, moderately, and marked stressful”) incidents are associated with cyclic responses in urinary cortisol and urinary neopterin levels. Each lag represents a time interval of 12 h. Coefficients (bars) reaching the upper or lower confidence limits (lines) are significant at *p*<.05. In **A**) negative incidents are followed by urinary cortisol concentration decreases at +lag 1 (12–24 h) and increases at +lag 6 (72–84 h). In **B**) negative incidents are preceded by urinary neopterin level increases at −lag 1 (0–12 h) and followed by urinary neopterin level decreases at +lag 4 (48–60 h).

Cross-correlational analyses showed that “somewhat, moderately, and marked stressful” incidents were associated with parallel increases in anxiousness/depressiveness (lag 0: +.298; *p* = 0.0009) and emotional irritation (lag 0: +.223; *p* = 0.013) followed by a decrease in irritation after 24–36 h (lag 2: −.180; *p* = 0.046). None of the other emotional states covered by the EWL (i.e. mental energy levels, general lethargy, extraversion/introversion, well-being) were found to co-occur with the emotionally negative incidents under study (data not shown). In regard to biochemical parameter levels, cross-correlational analyses showed that emotionally negative incidents were connected with cyclic fluctuations in both urinary cortisol and urinary neopterin concentrations. Urinary cortisol levels initially decreased 12–24 h after the occurrence of “somewhat, moderately, and marked stressful” incidents (lag 1: −.178; *p* = 0.048) and then increased a total of 72–84 h later (lag 6: +.224; *p* = 0.013). Urinary neopterin levels, on the other hand, first increased 0–12 h before “somewhat, moderately, and marked stressful” incidents occurred (−lag 1: +.185; *p* = 0.040) and then decreased a total of 48–60 h following such stressors (lag 4: −.181; *p* = 0.044). Figure 2 shows the cross-correlation functions of the stressor-cortisol (Fig. 2A) and stressor-neopterin (Fig. 2B) associations.

Next, we analyzed the impact of the two emotional states found to co-occur with “somewhat, moderately, and marked stressful” incidents (i.e. anxiousness/depressiveness, emotional irritation, see above) on biochemical parameter levels. Cross-correlational analyses revealed that neither parameter was followed by changes in urinary cortisol levels. However, increases in anxiousness/depressiveness were followed by decreases in urinary neopterin levels after 24–48 h (lag 2: −.183; *p* = 0.042) and, again, after 48–60 h (lag 4: −.177; *p* = 0.049). To examine this finding more closely, the subcategories of anxiousness/depressiveness, i.e. anxiousness, depressiveness and pensiveness, were each cross-correlated with urinary neopterin concentrations. These analyses showed that urinary neopterin levels were affected by both the subject's pensiveness and depressiveness, i.e. neopterin concentrations decreased 24–36 h following increases in pensiveness (lag 2: −.215; *p* = 0.017) and 48–60 h following increases in depressiveness (lag 4: −.221; *p* = 0.014). Anxiousness, on the other hand, had no impact on the course of urinary neopterin levels, nor was emotional irritation significantly related to urinary neopterin concentrations.

In a further step, we examined the impact of emotionally positive incidents on the course of urinary cortisol and urinary neopterin levels. Weekly interviews revealed one personally relevant emotionally positive theme dominating the subject's everyday life throughout the study period; we labeled this theme “educational and/or social accomplishment”. A total of 16 emotionally positive incidents related to “educational and/or social accomplishment” were identified in the interview transcripts. This category of positive incidents included choir rehearsals, final exam in public speaking course, mineralogy exam, weekly sessions for the current study, oral presentation at a scientific meeting, and preparatory discussions with her boss/thesis supervisor and another professor about the presentation.

Interviews revealed that such incidents were often precipitated by negative emotions (subject quotations translated from German: “nervous”, “under stress”, “uncomfortable”, “agitated”, “taxing”) and followed by positive emotions (“self-content”, “fun”, “moved”, “pleasant”, “satisfied”, “reaffirmed”, “high spirits”, “relieved”, “energized”, “happy”). Because of this co-occurrence of negative and positive emotions, six of the 16 emotionally positive incidents related to “educational and/or social accomplishment” (i.e. three of the nine weekly sessions for the current study, final exam in public speaking course, mineralogy exam, oral presentation at a scientific meeting) were also part of the time series of “somewhat, moderately, and marked stressful” incidents. In Table 2, two examples of emotionally positive incidents related to “educational and/or social accomplishment” are listed. One of these incidents, the presentation at a scientific meeting, was also rated as “marked stressful” (see also Table 1).

**Table 2:** Examples of emotionally positive incidents related to “educational and/or social accomplishment”.

**Day 11 (time unit 21): Choir rehearsal and performance in church**
During the preceding eight days, the subject had met her colleagues four times for choir practice. On this day, Boxing Day, she had a choir performance in her hometown church. A number of friends and her parents and siblings were present during the performance. After the performance, she was happy about its success and moved by the congratulations she received.
**Day 61 (time unit 121): Presentation in English at a scientific meeting ** ***(see *** ***Table 1*** *** for background information and negative part of the incident)***
In the subject's opinion, her presentation went rather well. Only a few questions were asked during the discussion part of her talk because the presenter before her had been “riddled” with questions, some of which also concerned the subject's topic and were thus already answered. Her boss and thesis supervisor, who was present during the presentation, winked at her and told her that she had done well, which made her happy. She did not join her colleagues to celebrate in the hotel, however, as she felt “dead tired” in the evening.

**Figure 3:**
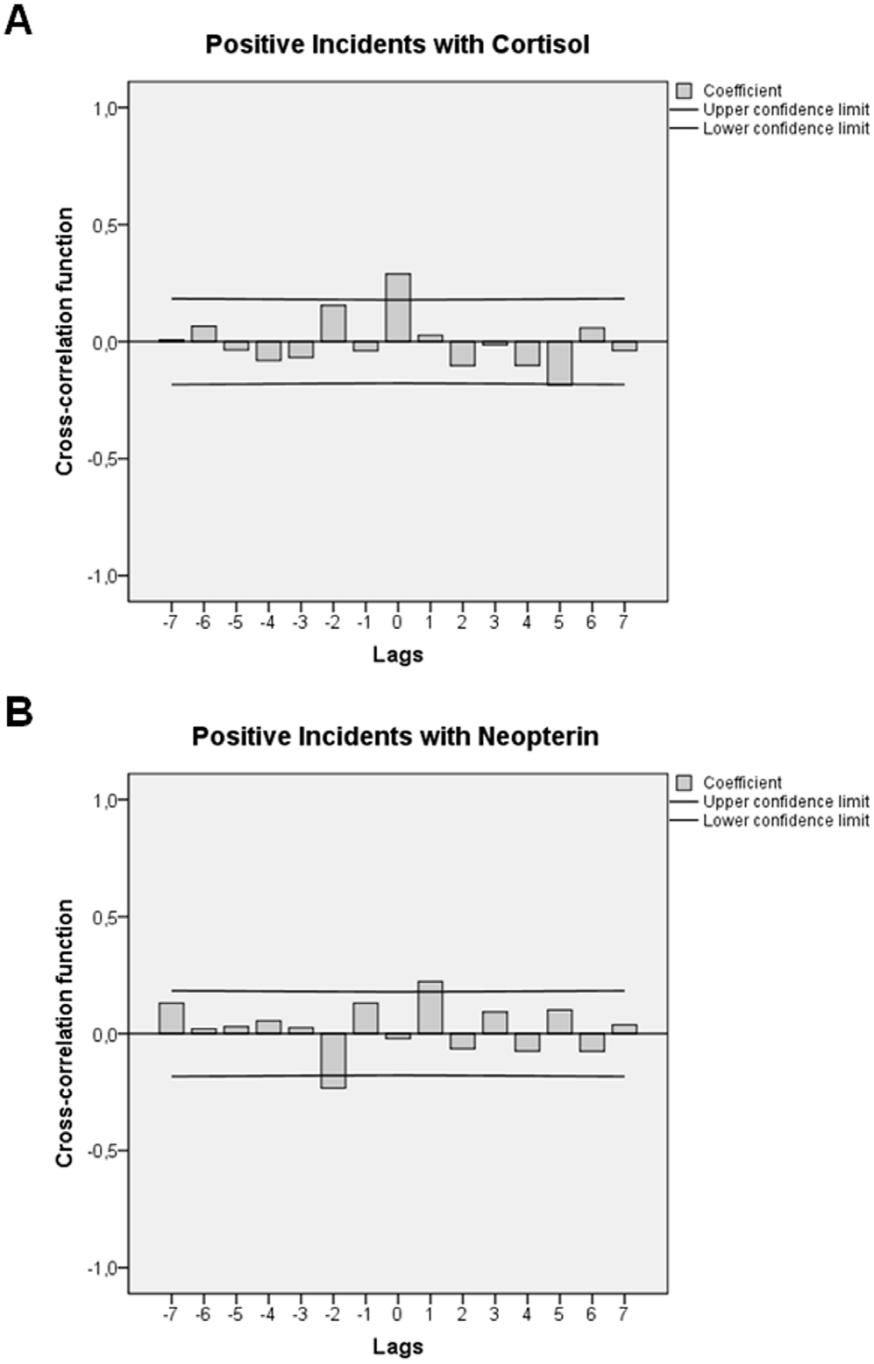
Adjusted cross-correlation functions indicating that positive incidents related to “educational and/or social accomplishment” are associated with cyclic responses in urinary cortisol and urinary neopterin levels. Each lag represents a time interval of 12 h. Coefficients (bars) reaching the upper or lower confidence limits (lines) are significant at *p*<.05. In **A**) positive incidents are followed by urinary cortisol concentration increases at ±lag 0 (0–12 h) and decreases at +lag 5 (60–72 h). In **B**) positive incidents are precipitated by urinary neopterin level decreases at −lag 2 (12–24 h) and followed by urinary neopterin level increases at +lag 1 (12–24 h).

Cross-correlational analyses showed that emotionally positive incidents related to “educational and/or social accomplishment” were not significantly paralleled by changes in any of the emotional states covered by the EWL. However, cross-correlating these incidents with urinary cortisol and neopterin concentrations showed cyclic fluctuations in both physiological parameters: Urinary cortisol levels first increased within 12 h following emotionally positive incidents related to “educational and/or social accomplishment” (lag 0: +.290; *p* = 0.001) and then decreased after a total of 60–72 h (lag 5: −.186; *p* = 0.039). Urinary neopterin levels, by contrast, initially decreased 12–24 h before such an emotionally positive incident occurred (−lag 2: −.233; *p* = 0.010) and increased a total of 12–24 h following the incident (lag 1: +.222; *p* = 0.014). Figures 3A and 3B show the corresponding cross-correlograms.

None of the other variables gathered in this study significantly affected the cross-correlational findings presented above.

## Discussion

The findings of this integrative single-case study on a healthy woman illustrate the type of additional information that can be attained when complex data sets based on time series and interviews are handled appropriately in stress research under real-life conditions. Specifically, four main findings emerge: 1) The time intervals between onset of emotionally meaningful everyday incidents and stress system response were rather long, i.e. up to 72–84 h for urinary cortisol increases and 48–60 h for urinary neopterin decreases associated with negative incidents and emotions, and up to 60–72 h for urinary cortisol decreases and 12–24 h for urinary neopterin increases associated with positive emotional experiences; 2) the pattern of stress system parameter changes in response to emotionally meaningful incidents was biphasic; 3) negative incidents sharply contrasted with positive incidents in terms of their effects on stress system functioning; 4) the functioning of the healthy stress system under study showed the opposite behavior of that found in our earlier integrative single-case studies on patients with SLE [48]–[50].

According to system-theoretical thinking, the stress-reaction process in humans can be seen as a complex psychobiological feedback circuit, in which multi-phase appraisals of the stressor and its meaning for the individual, and physiological and psychological responses that allow coping with the situation continuously interact and regulate each other via feedback mechanisms [69], [70]. Study designs in stress research, therefore, may be more representative [71] when they investigate highly complex phenomena such as the stress-reaction process in everyday life. When one compares the results of the current study with those of other investigations on stress system functioning, it appears that as the representativeness of the study approach increases the temporal delay between the onset of stress and stress system response increases; the pattern of stress system response becomes more complex; and the stress system response to emotionally negative and positive incidents becomes more differentiated.

In laboratory studies – in which stressors usually do not match the intensity or duration of real-life stressors [72] and in which the stress system activity of individuals is tested under spatial and temporal conditions fundamentally dissimilar to peoples' natural environment (e.g. strict control of external influences, temporal limitation to a few hours) – the temporal delay between stress onset and increase in stress system activity may be 20–40 min for cortisol [9], [15] and 30 min to 2 h for cellular immune parameters such as IL-6, IL-1β, IL-1Ra, and TNF-α [17]–[20]. By contrast, in naturalistic studies applying EMA and ESM designs stress system responses have been observed that clearly last longer than in laboratory research, i.e. 1 day for cortisol [36]–[38] and 1–2 days for sIgA changes [39].

In order to ensure that stress research is as representative as possible, integrative single-case studies may be better adapted to the everyday reality than EMA and ESM studies in that they i) use time-series analysis of continuous and extensive temporal data sets, ii) apply qualitative methods to determine the personal meaning of incidents, iii) rely on a fixed schedule for daily questionnaires, and iv) explicitly focus on the single individual. Based on these methodological specifics, the present study on a healthy woman showed that the stress system response process can be considerably longer and more complex and differentiated than EMA and ESM studies have suggested.

Specifically, we demonstrated that emotionally meaningful everyday stressors (“somewhat”, “moderately” and “marked” stressful) were followed by cyclic or biphasic responses in both urinary cortisol and urinary neopterin concentrations. Urinary cortisol levels first decreased 12–24 h after the occurrence of a stressful incident and then increased after a total of 72–84 h. Urinary neopterin levels, on the other hand, first increased 0–12 h before stressors occurred and then decreased a total of 48–60 h following stressors. The finding that the first phase of the cyclic urinary neopterin response happened 0–12 h before the occurrence of a stressful incident might be explained by incident anticipation [49], [50], as the subject was aware of many such incidents days or even weeks beforehand. In addition, in this study, increases in pensiveness and depressiveness were followed by decreases in urinary neopterin levels, 24–36 h and 48–60 h later, respectively. The ultimate suppression of neopterin levels following both negative incidents and negative emotional states in a healthy individual is in line with findings from Dunbar et al. [73], who showed that examination stress in healthy students was associated with a decrease in the urinary neopterin/creatinine ratio. However, as to the stress-related cyclic pattern of urinary cortisol response seen in the current study, no comparable empirical findings are available in the literature, although speculations from meta-analyses on the biphasic or cyclic nature of the cortisol response to chronic stressors have been made [11]. Thus, it remains unclear whether the increase in urinary cortisol concentration in the second phase of the biphasic stress response process observed in this study corresponds to the stress-induced cortisol increase usually seen in healthy individuals [13].

It should be pointed out that in this study 23 cross-correlational analyses were performed in total, and that most of the significant effect sizes from these analyses were relatively small (mean r = .213) and only reached statistical significance at the .05 level (mean *p* = 0.026). Some readers might therefore question the validity of our findings, especially since we did not adjust p-values upward to reduce the chance of incorrectly declaring statistical significance in multiple cross-correlations [62]. However, several specifics related to the time-series approach used in this study should be kept in mind when interpreting the statistical findings. For example, we applied adjusted cross-correlational analyses to avoid spurious cross-correlations, i.e. we separated serial dependencies (e.g. day-night rhythm, menstrual cycle) from residuals by modeling time series, and used these residuals for cross-correlational analyses. This is a rather conservative approach which lowers effect sizes and makes it harder to achieve significant findings compared to unadjusted cross-correlational analysis [41]. In addition, our exploratory study showed that an exclusive focus on whether single cross-correlation coefficients reach significance may not be sufficient for a proper interpretation of findings from cross-correlational analyses. Rather, to be more effective in this regard, the temporal pattern of coefficients (e.g. cyclic) in cross-correlation functions should be analyzed carefully. This is in line with the notion that the discovery of patterns is fundamental in time-series analysis [41] and that for their detection and recognition, descriptive rather than inferential statistics is required [64].

In this study, we used both emotionally meaningful negative and positive incidents to test stress system functioning under real-life conditions. With regard to the differential effects of emotionally positive and negative incidents on peripheral cortisol and immune parameter levels in healthy individuals, findings from conventional group studies have been equivocal. In laboratory studies, stress system activities in response to positive and negative emotion induction have been shown to be similar [74], [75]. On the other hand, both laboratory studies [76], [77] and naturalistic studies applying the EMA or ESM paradigm have found either no association between emotionally positive experiences and stress system activity [36], [78]–[80], or have noticed a sharp contrast in stress system activity depending on whether experiences were emotionally positive or negative [39], [81]–[86]. It appears that such inconsistencies may be due to the varying degrees of representativeness of study designs.

The present integrative single-case study revealed sharp contrasts in stress system activities related to the occurrence of emotionally meaningful positive or negative incidents. Moreover, this study identified cyclic patterns of stress system activity in response to emotionally meaningful positive incidents similar to those associated with emotionally meaningful negative incidents. Specifically, it was shown that whenever the subject experienced emotionally positive incidents related to the personally relevant theme “educational and/or social accomplishment”, urinary cortisol levels first increased within 12 h and then decreased a total of 60–72 h later; urinary neopterin levels, in turn, first decreased 12–24 h before such positive incident occurred and then increased a total of 12–24 h after the incident. As the subject was aware of most such incidents days or even weeks beforehand, it can be assumed – similar to the stressor-neopterin association in this subject (see above) – that the neopterin level change before the occurrence of emotionally positive incidents was due to anticipation [49], [50]. As with the biphasic response patterns of urinary cortisol and urinary neopterin to everyday stressors, there are no comparable findings in the literature regarding cyclic patterns of stress system parameters in response to emotionally positive incidents in healthy individuals. In order to interpret the cyclic response patterns seen in this study, it is useful to look at the dynamic and functional characteristics of the stress process, known mostly from animal studies.

In animal studies, a single experience of IS can activate a cascade of various neurobiological processes, some of which take a few hours to return to baseline, others days (e.g. more than 96 h or 4 days for corticosterone increase in [28]) or even weeks [25]. Moreover, the immune response to an initial infection, i.e. a physical stressor, occurs in three phases, the immediate (0–4 h), the early (4–96 h), and the late (>96 h) phase, where the first two phases rely on the innate immune system and the third phase on the adaptive immune system [87]. These stress system responses not only operate over multiple time scales but also regulate each other through complex networks of control systems, feedback loops, and other regulatory mechanisms [88]. For example, within the so-called immuno-neuro-endocrine network, HPA axis activation and cortisol release counter-regulate stress-mediated increases in cellular immune activity through feedback inhibition in order to protect initial immune reactions from overshooting [89], [90]. It has also been shown that such negative feedback loops – typically occurring in the later stages of the stress response process [26] – work over long time scales. Glucocorticoid feedback, for example, can be differentiated into fast (within 30 min) and delayed feedback (after 60 min or more), and the latter can be further subdivided into intermediate (2–10 h) and slow feedback (12 h or more) [5].

Taking these observations from experimental studies into account, the incident-related long-term cyclic response patterns in urinary cortisol and neopterin levels seen in our naturalistic study on a healthy woman could be interpreted as follows: While cortisol decreased in the initial stages of the response to emotionally negative incidents, it may have been slowly counter-regulated in the later stages, resulting in a long-term increase. Similarly, cortisol first increased and neopterin first decreased in response to emotionally positive incidents, then being slowly counter-regulated to ultimate cortisol decreases and neopterin increases, respectively.

It remains unclear whether these cyclic responses are direct consequences of the incidents or whether they can be considered secondary- or higher-order responses caused by interaction and feedback between appraisals and psychological as well as physiological coping efforts [25], [70]. Indeed, the significant cross-correlations between negative emotional states and neopterin levels seen in this study and findings from a recent laboratory study applying time series analysis on psychoendocrine data [21] support the notion that psychophysiological stress responses are more closely coupled over time than commonly assumed. Nevertheless, the psychoimmunological and psychoendocrinological cascades shown in our study are far from understood, and further studies are needed to systematically investigate the extent to which the various psychological and physiological parts of the stress response process contribute to the patterns seen here.

To what extent are the findings of this study similar or dissimilar to those of previous studies of our working group? In other words, do the current results from a healthy woman replicate findings from previous studies on SLE patients [44], [48]–[50]? These questions may be approached by considering this study as part of a multiple case study according to Yin's definition [91]. Yin [91] has proposed two kinds of replication in multiple-case studies: i) literal replication, in which a case study predicts results similar to findings of previous case studies, and ii) theoretical replication, in which a case study leads to findings contrasting with results of previous case studies but for predictable reasons. We started with three studies on patients with SLE and were able to *literally* replicate our first findings, i.e. findings from Case 2 were quite similar to results from Case 3 [48] (see Introduction). Case 1 [44] used a different, somewhat simpler design and thus cannot be compared with Cases 2 and 3.

Findings from the present integrative single-case study on a healthy woman (Case 4) show both similarities and dissimilarities to the findings from our studies on SLE patients. Similarities pertain to the complex stress system activity patterns in response to emotionally meaningful everyday incidents, i.e. the long temporal intervals between the occurrence of incidents and stress system response; the biphasic stress-reaction pattern; the anticipatory stress-system reactions; and the sharp contrast in stress system activity in response to positive and negative incidents [48]–[50]. Dissimilarities concern the directions of stress-system response patterns in the healthy woman under study, which are exactly in contrast to those demonstrated in our studies on SLE patients [48]–[50]. These contrasting responses are principally in line with recent evidence from experimental and clinical studies on stress system functioning: In a normal stress system, stress is typically associated with increased cortisol release and decreased cellular immune function [9], [10], [13]. HPA axis hypofunction, on the other hand, which is common in diseases like SLE, is characterized by stress system responses opposing those seen in healthy subjects, i.e. decreases in cortisol release (i.e. hypocortisolism) and increases in cellular immune activity [8], [45], [46], [92]–[94]. Thus, a closer look at the literature on stress system functioning reveals that, although contrasting, our current findings could have been predicted from our previous studies on SLE patients. In other words, the current results on a healthy person may be seen as a *theoretical* replication according to Yin [91].

The limitations of this study concern in particular its exploratory character, associated with several design-related (e.g. n = 1) and statistical specifics (e.g. no alpha adjustment for multiple tests), which render the findings preliminary. Further replication is thus required. Moreover, future studies could profit from a more differentiated evaluation with regard to both psychological issues (e.g. intensity rating of positive incidents; applying an additive model of stress) and biochemical issues (e.g. measuring direct markers of cytokine activity). Also, the statistical method of evaluating psychosomatic interdependencies could be expanded in future investigations to include multivariate time-series analyses in order to identify psychophysiological feedback mechanisms and other indicators of psychosomatic complexity [95].

Besides these limiting factors, our experience with integrative single-case studies suggests that in biopsychosocial research the appropriate consideration of “time” (by using time series analysis) and “meaning” (by using qualitative methods) may lead to insights into the dynamic characteristics of the normal stress-reaction process that are not achievable by less representative approaches, such as laboratory studies or those applying the EMA or ESM design. Moreover, the existence of biphasic stress system responses and other features of psychosomatic complexity may at least in part explain the inconsistencies in conventional stress research and the poor lab-to-life generalizability [13], [96], [97]. Life is definitively too complex to grasp with snapshot-like investigations of psychological and physiological data sets that are averaged across individuals. Rather, investigating the unique internal constellation of single variables, their specific dependencies and correlations over time, and, consequently, challenging the practice of generalization based on simple data aggregation appears to be a valid route in stress research that is worthy of further development.
